# Non-operative treatment of children with simple appendicitis: long-term follow-up (5 years) in a prospective cohort study

**DOI:** 10.1093/bjs/znaa052

**Published:** 2021-02-01

**Authors:** M . Knaapen, J H Van der Lee, E L Gaillard, H A Cense, K H in ‘t Hof, C M F Kneepkens, M H Wijnen, H A Heij, R Bakx, L W E van Heurn, R R Gorter

**Affiliations:** 1 Department of Paediatric Surgery, Emma Children’s Hospital, Amsterdam UMC, University of Amsterdam and Vrije Universiteit Amsterdam, Amsterdam, the Netherlands; 2 Paediatric Clinical Research Office, Emma Children’s Hospital, Amsterdam UMC, University of Amsterdam, Amsterdam, the Netherlands; 3 Knowledge Institute of the Dutch Association of Medical Specialists, Utrecht, the Netherlands; 4 Department of Surgery, Red Cross Hospital, Beverwijk, the Netherlands; 5 Department of Surgery, Flevoziekenhuis, Almere, the Netherlands; 6 Department of Paediatric Gastroenterology, Amsterdam UMC, University of Amsterdam and Vrije Universiteit Amsterdam, Amsterdam, the Netherlands; 7 Department of Paediatric Surgery, Princess Maxima Centre, Utrecht, the Netherlands

## Abstract

Long-term results after non-operative treatment for children with simple appendicitis seem promising, possibly avoiding appendicectomy in 70 per cent of children after a median follow-up of 5 years. The need for delayed appendicectomy more than 2 years after the initial treatment is rare (0–5 per cent) and no complications occurred past 1 year, including children who underwent delayed appendicectomy.

*Dear Editor*

Pilot studies have shown that non-operative treatment with antibiotics (NOT) has a high initial success rate in children with simple appendicitis^1^^,^^2^. However, few data exist on the long-term outcomes, which are particularly relevant as patients may over time undergo delayed appendicectomy for recurrent appendicitis or abdominal complaints. After 1 year, it is estimated that approximately three of four children with NOT for simple appendicitis have avoided an appendicectomy^1^.

Here, the authors re-evaluated 49 children who previously received NOT for acute simple appendicitis in a feasibility study for the APAC (Antibiotics *versus* Primary Appendectomy in Children) trial, an ongoing randomized trial comparing NOT with appendicectomy in children with simple appendicitis; its methods, short- and medium-term results were published in 2015^2^ and 2018^3^. Children aged 7–17 years with ultrasound-confirmed simple appendicitis without a faecalith were treated with intravenous antibiotics for 48–72 h, continued orally at home for 5 days. In the event of clinical deterioration, insufficient recovery or recurrent appendicitis, appendicectomy was performed. To assess long-term outcomes after NOT, data on delayed appendicectomies and complications were collected during telephone follow-up interviews (October 2019); in addition, electronic health records, including histopathological reports, were investigated. All events were scored separately by three authors according to type, severity, and relationship to appendicitis or its treatment, using preset definitions.

In total, 47 of 49 children could be contacted for follow-up at a median of 5.4 (range 3.9–7.1) years after initial treatment. Appendicectomy had not been performed in 33 of 47 children (70 (95 per cent c.i. 56 to 81) per cent) (*Fig. 1*). Of the 14 children who did undergo appendicectomy, four (9 (3 to 20) per cent) were classified as non-responders (failure of NOT within 7 days after start of treatment), and 10 (21 (12 to 35) per cent) as having delayed appendicectomy. Histopathological examination after the delayed appendicectomies showed simple appendicitis (7), non-inflamed appendices (2), and chronic inflammation with fibrosis (1). In none of the children who had delayed appendicectomies was complex appendicitis diagnosed. No complications occurred past 1 year, including children who underwent delayed appendicectomy.

**Fig. 1:**
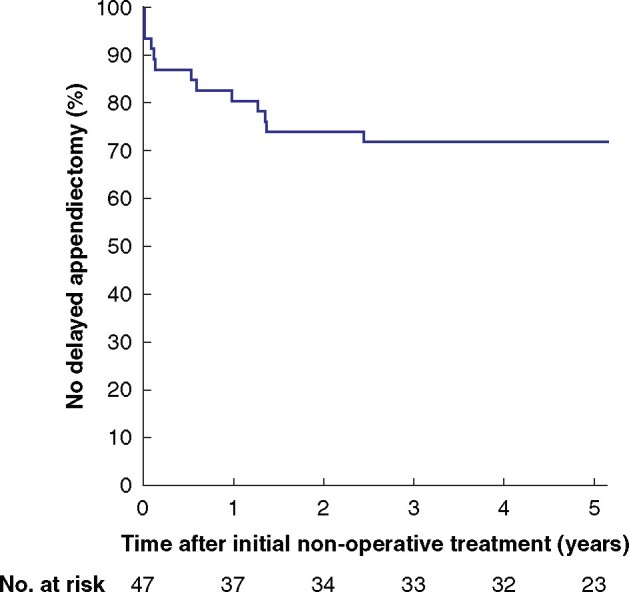
Kaplan–Meier curve for time to delayed appendicectomy after non-operative treatment failure

The long-term rates of delayed appendicectomy are similar to those in two^4^^,^^5^ of three studies that reported long-term outcomes after NOT in children. A third study^6^, a 5-year follow-up of a pilot RCT, reported a higher total appendicectomy rate, with 11 of 24 children randomized to NOT undergoing appendicectomy. However, histopathological examination showed acute appendicitis in only four of the 24 children randomized to NOT. The authors suggested that this could be the result of the novelty of NOT resulting in more liberal indications for surgery^6^. In the present cohort, only one patient had an appendicectomy after more than 2 years, which is similar to findings of the previously mentioned studies^4–6^, indicating that the need for delayed appendicectomy more than 2 years after the initial treatment is rare (0–5 per cent).

The goal of NOT is to prevent appendicectomy, which would otherwise be done in all children. The exposure to anaesthesia, surgical stress, and potential complications are avoided in most patients. However, the authors do not consider NOT to be a replacement for appendicectomy, but rather part of a step-up approach, preserving surgery for children who actually need it. They are currently awaiting the results of several ongoing RCTs for definitive evidence on the medium-term (1 year) efficacy of NOT compared with surgery, including other relevant outcomes, such as quality of life, disability days, and costs. From the results of the present study it can be concluded that the long-term results after NOT for children with simple appendicitis seem promising, possibly avoiding appendicectomy in 70 per cent of children after a median follow-up of 5 years.

*Disclosure.* The authors declare no conflict of interest.
